# Opioid treatment program safety measures during the COVID-19 pandemic: a statewide survey

**DOI:** 10.1186/s12913-022-07832-7

**Published:** 2022-03-30

**Authors:** Sachini Bandara, Hannah Maniates, Eric Hulsey, Jennifer S. Smith, Ellen DiDomenico, Elizabeth A. Stuart, Brendan Saloner, Noa Krawczyk

**Affiliations:** 1 Department of Mental Health, Johns Hopkins Bloomberg School of Public Health, Baltimore, MD 21205 USA; 2 Department of Health Policy and Management, Johns Hopkins Bloomberg School of Public Health, Baltimore, MD 21205 USA; 3 Vital Strategies, 100 Broadway 4th Floor, New York, NY 10005 USA; 4 Pennsylvania Department of Drug and Alcohol Programs, Harrisburg, PA 17110 USA; 5 Center for Opioid Epidemiology and Policy, Department of Population Health, NYU Grossman School of Medicine, New York, NY 10016 USA

**Keywords:** Opioid use disorder, Substance use disorder treatment, COVID-19

## Abstract

**Background:**

Opioid treatment programs (OTPs) serve as daily essential services for people with opioid use disorder. This study seeks to identify modifications to operations and adoption of safety measures at Pennsylvania OTPs during the COVID-19 pandemic.

**Methods:**

A 25-min online survey to clinical and administrative directors at all 103 state-licensed OTPs in Pennsylvania was fielded from September to November 2020. Survey domains included: 1) changes to services, client volume, hours and staffing during the COVID-19 pandemic 2) types of services modifications 3) safety protocols to reduce COVID-19 transmission 4) challenges to operations during the pandemic.

**Results:**

Forty-seven directors responded, for a response rate of 45%. Almost all respondents reported making some service modification (96%, *n* = 43). Almost half (47%, *n* = 21) of respondents reported reductions in the number of clients served. OTPs were more likely to adopt safety protocols that did not require significant funding, such as limiting the number of people entering the site (100%, *n* = 44), posting COVID-safety information (100%, *n* = 44), enforcing social distancing (98%, *n* = 43), and increasing sanitation (100%, *n* = 44). Only 34% (*n* = 14) of OTPS provided N95 masks to most or all staff. Respondents reported that staff’s stress and negative mental health (86%, *n* = 38) and staff caregiving responsibilities (84%, *n* = 37) during the pandemic were challenges to maintaining OTP operations.

**Conclusion:**

OTPs faced numerous challenges to operations and adoption of safety measures during the COVID-19 pandemic. Funding mechanisms and interventions to improve adoption of safety protocols, staff mental health as well as research on patient experiences and preferences can inform further OTP adaptation to the COVID-19 pandemic and future emergency planning.

**Supplementary Information:**

The online version contains supplementary material available at 10.1186/s12913-022-07832-7.

## Background

The novel coronavirus 2019 (COVID-19) pandemic fundamentally shifted life, including placing enormous strain on health care systems and services. Because COVID-19 is highly contagious and transmitted via exposure to infectious respiratory aerosols, states, localities and health care systems undertook several policies to reduce the spread of disease, including masking mandates, stay-at-home orders and altering the ways in which health care services are provided to enhance physical distancing [1–3].

Important questions remain on how specialized behavioral health services, such as those targeting people with substance use disorders, adapted to the pandemic circumstances. This is especially important as the COVID-19 emergency is occurring in the context of an overdose crisis in the United States (US) [4, 5]. Provisional data from the U.S. Centers for Disease Control and Prevention estimate that in 2020, over 93,000 individuals died from drug overdose, a staggering increase from the already elevated 2019 data of over 72,000 deaths [6]. Almost 70,000 overdose deaths in 2020 were opioid-related [6]. Non-fatal overdoes rates also rose in 2020 [7, 8]. During this time, individuals with opioid use disorder were also at increased risk for COVID-19-related health complications [4]. Because these two public health crises are intertwined, it is necessary to understand how the pandemic impacted the provision of opioid use disorder treatment and what COVID-19 risk mitigation efforts these treatment providers adopted.

Opioid agonist treatments, like buprenorphine and methadone, are the gold-standard treatments for opioid use disorder, with extensive evidence showing they reduce mortality and improve treatment outcomes [9–13]. However, people with opioid use disorder must take these medications daily and accessing these medications in the U.S. is difficult and highly regulated [10]. This in part contributes to low uptake of opioid agonist treatment and disparities in treatment use by race and socioeconomic status [10]. Under U.S. federal law, methadone to treat opioid use disorder must be dispensed in licensed specialty clinics, called opioid treatment programs (OTPs), that have a license from the U.S. Drug Enforcement Administration. In addition to methadone, physicians working at OTPs can also prescribe or dispense buprenorphine treatment [10]. In the U.S., many OTPs operate via private for-profit or non-profit organizations outside of mainstream health services and larger health care systems. Federal and state regulations require patients make near-daily visits to OTPs in the first 90 days and at least weekly visits in the first year of treatment, and often require participation in adjunct services, such as psychosocial counseling and drug screening [14, 15]. Because most patients are required to visit what are often crowded OTPs daily or near daily to receive services under direct observation, these programs are uniquely susceptible to safety issues related to the COVID-19 pandemic. In an attempt to alleviate some of these COVID-19 transmission concerns, federal and state agencies issued several guidance documents allowing for enhanced use of telehealth services and increased the maximum number of days of take-home methadone for patients to self-administer at home (14 days for less stable patients and 28 days for highly stable patients, based on risk criteria established over a decade ago) [16].

A small but growing body of research has documented changes to OTP practices regarding opioid agonist treatment, particularly in response to these regulatory changes. These studies find increased use of take-home medications, increased provision of psychosocial services via tele-health and mixed results on urine drug screening practices [17–23]. However, less is known about how the pandemic altered OTP operations and what program-level safety protocols were made to mitigate COVID-19 risk. A case study of three OTPs by Quiñones et al. found increased use of telemedicine to separate staff and clients within the clinic, changes in the number of days that services were provided and adoption of some safety measures to encourage physical distancing [19]. A national survey of 142 OTPs conducted by the U.S. Department of Health and Human Services early in the pandemic reported changes to OTP service levels, the adoption of various personal safety measures, physical alterations to facilities and challenges related to maintaining supplies of personal protective equipment and medical supplies [23]. To further understand these issues, we fielded a statewide survey of OTPs in Pennsylvania, a state with one of the highest overdose mortality rates [24], and identified safety and operational changes made by OTPs in the state during the COVID-19 pandemic.

## Methods

### Survey sample and administration

The survey sample included all OTPS licensed by the Pennsylvania Department of Drug and Alcohol Programs, totaling to 103 OTPs. Respondents included clinical or administrative directors, who were chosen because of their leadership and ability to speak on decision-making around OTP operations and safety protocols compared to other OTP staff. In September 2020, staff from the Pennsylvania Department of Drug and Alcohol Programs identified and contacted a director for each OTP via email, inviting them to participate in a voluntary online research survey. Respondents received 2 subsequent reminder emails before data collection stopped in November 2020. The 25-min online survey was administered via Qualtrics. This study was approved by the Johns Hopkins Bloomberg School of Public Health Institutional Review Board.

### Survey domains

The survey included six domains: 1) any change to services and operations, client volume, hours and staffing during the COVID-19 pandemic, 2) specific types of services modifications, 3) specific types of safety protocols adopted to reduce COVID-19 transmission, 4) challenges to operations during the pandemic, 5) specific types of modifications to medication treatment services, and 6) attitudes on medication treatment service and regulatory changes (see Additional File 1 for survey instrument). This analysis focuses on the first four domains; findings from domains related to medication treatment are reported in a separate analysis by Krawczyk et al. [18].

To measure changes to services, client volume, hours and staffing during the pandemic, respondents were asked what services were provided pre-pandemic and if any services were modified. In separate measures, respondents were asked if full-time equivalent staff, client volume or hours were not changed, reduced or increased. For each type of service provided pre-pandemic, respondents were asked if OTPs increased or decreased the volume of this service, switched the service to a remote or virtual platform, changed the schedule or made no change. Respondents were allowed to endorse multiple options for each of these questions.

Safety protocols were assessed in two ways. First, respondents were presented with a list of safety protocols and asked to endorse what measures were used by the OTP to manage client and staff risk for COVID-19 transmission. Second, to measure access to personal protective equipment among the staff, we asked respondents to list if all, most, some or no staff had access to the following equipment: cloth/surgical masks, gloves and N95 masks. The percentage of respondents that endorsed either all or most staff were collapsed together and reported.

Finally, to measure challenges to operations during the pandemic, respondents were presented with a list of potential challenges and were asked to rate how much they agreed or disagreed that the challenge posed a problem for the organization during the pandemic. Respondents rated their agreement on a 4-point Likert scale (Strongly agree, somewhat agree, somewhat disagree, or strongly disagree). The percentage of respondents that endorsed either strongly or somewhat agree were collapsed together and reported.

### Statistical analysis

We calculated descriptive statistics for each survey item. Because respondents were only asked about services and operations modifications for activities they engaged in prior to the pandemic, the number of responses varied by question. Response percentages were calculated using a denominator of the total number of respondents that answered the question, which are noted in the exhibits.

## Results

Of the 103 OTP directors in the sample, directors from 47 OTPs completed the survey for a response rate of 45%. The size of respondent OTPs varied, with a median 264 clients (IQR 180–428) and a median of 21 full-time equivalent staff (IQR 15–40). Sixty-four percent of OTPs were for-profit, rather than not-for-profit organizations. Of the respondents that answered questions about clientele race and ethnicity (*n* = 45), 11% reported clientele being comprised of some White Non-Hispanic clients, 64% reported clientele being comprised of mostly White Non-Hispanic clients, and 24% reported clientele being comprised of nearly all White Non-Hispanic clients. Seventy-one percent reported clientele being comprised of some Black Non-Hispanic clients and 9% reported clientele being comprised of mostly Black Non-Hispanic clients. Sixty percent reported clientele being comprised of some Hispanic clients and 2% reported clientele being comprised of mostly Hispanic clients.

Prior to COVID-19, 93% (*n* = 41) of respondents offered methadone maintenance, 52% (*n* = 23) offered buprenorphine maintenance, 39% (*n* = 17) offered extended-release naltrexone maintenance, 11% (*n* = 5) offered methadone detoxification/tapering and 9% (*n* = 4) offered buprenorphine detoxification/tapering. Of OTPs that offered buprenorphine treatment, 71% prescribed buprenorphine by a waivered provider to be filled at a community pharmacy and 58% dispensed buprenorphine on site.

### Changes to services, client volume, hours and staffing

**Fig. 1:**
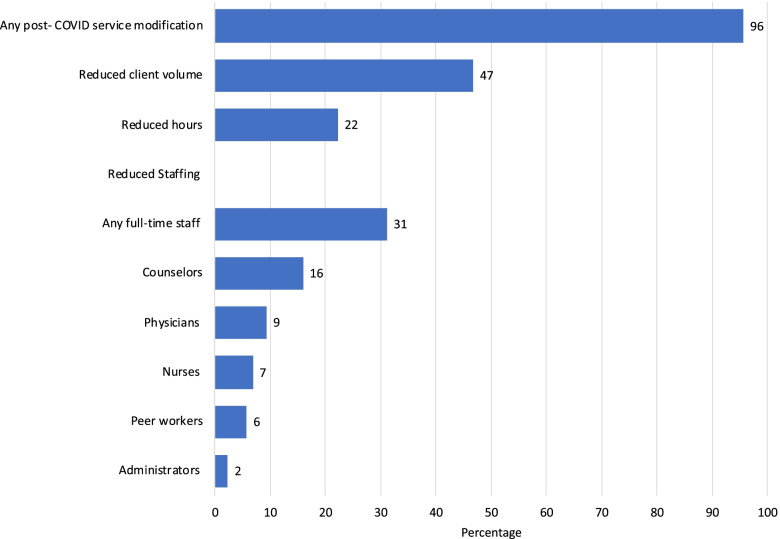
Changes to client volume, hours and staffing in Pennsylvania OTPs during the COVID-19 pandemic. Note: All respondents did not answer every question. The total number of respondents who answered the question for each measure is noted by each row

Ninety-six percent (*n* = 43) of respondents made at least one modification to services or operations during the COVID-19 pandemic as reported in Fig. 1. Almost half of respondents reported reductions in the number of clients served (47%, *n* = 21), and fewer reported reduced hours (22%, *n* = 10) or reduced full-time staff (31%, *n* = 14). Respondents were most likely to report reducing counselors (16%, *n* = 7), followed by physicians (9%, *n* = 4), nurses (7%, *n* = 3), peer workers (6%, *n* = 2) and administrators (2%, *n* = 1).

### Types of service modifications

Changes in service delivery by type of services are reported in Table 1. Thirteen percent (*n* = 5) increased the amount of medications for opioid use disorder (MOUD) services provided, 5% (*n* = 2) decreased amount of MOUD services provided, 13% (*n* = 5) switched MOUD services to remote or virtual platforms, 18% (*n* = 7) made changes to the MOUD service schedule, and 65% (*n* = 26) made no change to MOUD services.

**Table 1:** Services modifications in Pennsylvania OTPs during the COVID-19 pandemic

	**Percent (n)**		
	**Increased Services**	**Decreased Services**	**Switched to remote services**
**Medications for opioid use disorder** (MOUD) (*n* = 40)	13	5	13
	(5)	(2)	(5)
**Walk-in/ Same day treatment initiation (*n* = 21)**	5	19	5
	(1)	(4)	(1)
**Non-MOUD services**			
On-site case management and social services (*n* = 18)	6	17	22
	(1)	(3)	(4)
Referral out to case management and social services (*n* = 27)	11	26	0
	(3)	(7)	(0)
Individual counseling (*n* = 42)	7	7	55
	(3)	(3)	(23)
Group counseling (*n* = 38)	3	18	37
	(1)	(7)	(14)
Drug screening (*n* = 35)	9	23	0
	(3)	(8)	(0)
Infectious disease services (*n* = 14)	7	29	0
	(1)	(4)	(0)
Naloxone distribution (*n* = 25)	36	8	4
	(9)	(2)	(1)

Respondents were asked if they modified services, only for the services that were reported as being provided pre-COVID. Respondents could endorse more than one category for change to each service type

Among OTPs that offered walk-in or same day treatment initiation pre-pandemic, 5% (*n* = 1) increased, 33% (*n* = 7) decreased these services and 52% (*n* = 11) made no change. Among OTPs that offered on-site case management and social services pre-pandemic, 17% (*n* = 3) decreased these services, 22% (*n* = 4) moved this service to remote or virtual platforms and 56% (*n* = 10) made no change. Among OTPs that offered referrals out to case management and social services, 26% (*n* = 7) decreased these services and 67% (*n* = 18) made no change.

We then examined changes to counseling services. Among OTPs that offered individual counseling services pre-pandemic, 7% (*n* = 3) increased these services, 7% (*n* = 3) decreased these services, 55% (*n* = 23) moved these services to virtual platforms and 33% (*n* = 14) made no change. Among OTPs that offered group counseling services pre-COVID-19, 50% (*n* = 19) decreased these services, 37% (*n* = 14) moved group counseling services to virtual platforms, and 18% (*n* = 7) made no change.

With regards to other screening and health services, 26% (*n* = 9) of OTPs that did drug screenings pre-COVID decreased the amount of screenings conducted and 66% (*n* = 23) made no change. Among OTPs that provided infectious disease services pre-COVID-19, 29% (*n* = 4) decreased these services and 64% (*n* = 9) made no change. Of OTPs that distributed naloxone pre-COVID-19, 36% (*n* = 9) increased naloxone distribution, and 8% (*n* = 2) decreased distribution and 56% (*n* = 14) made no change.

### Safety protocols

OTPs engaged in a variety of safety protocols to reduce COVID-transmission as reported in Table 2. All OTPs (*n* = 44) limited the number of people entering the site at a time, implemented additional sanitation precautions beyond handwashing stations such as wiping surfaces, and posted safety information on flyers or the organizational website. Almost all OTPs enforced social distancing (98%, *n* = 43), had handwashing stations on site (96%, *n* = 42), increased space in the waiting areas (93%, *n* = 41), required face coverings (89%, *n* = 39), expanded telehealth services (84%, *n* = 37), or screened individuals for COVID-19 symptoms (80%, *n* = 35). Less than two thirds of OTPs had staggered visits (61%, *n* = 27), added physical barriers (57%, *n* = 25), referred patients elsewhere for some services (48%, *n* = 21), or created triage areas to avoid lines (39%, *n* = 17).

**Table 2:** Safety protocols and personal protective equipment access in Pennsylvania OTPs during the COVID-19 pandemic

**Safety Protocols Adopted**	**% Adopted**	**n**
Limit number of people entering site	100	44
Additional sanitation precautions	100	44
Safety info posted	100	44
Enforced social distancing for patients	98	43
Handwashing/sanitizing stations on site	96	42
Increased space in waiting area	93	41
Required face coverings	89	39
Expanded telehealth services	84	37
Screening for COVID-19 symptoms	80	35
Staggered visits	61	27
Added physical barriers	57	25
Referred patients elsewhere for some services	48	21
Created triage areas to avoid lines	39	17
**Staff Personal Protective Equipment Access**	**% endorse that all or most staff have access**	**n**
Cloth/Surgical Masks	100	43
Gloves	98	42
N95 Masks	34	14

Regarding personal protective equipment, all OTPs provided cloth or surgical masks to most or all staff (*n* = 43), 98% of OTPs provided gloves to most or all staff (*n* = 98), and 34% provided N95 masks to most or all staff (*n* = 14).

### Challenges to operations during the pandemic

**Fig. 2:**
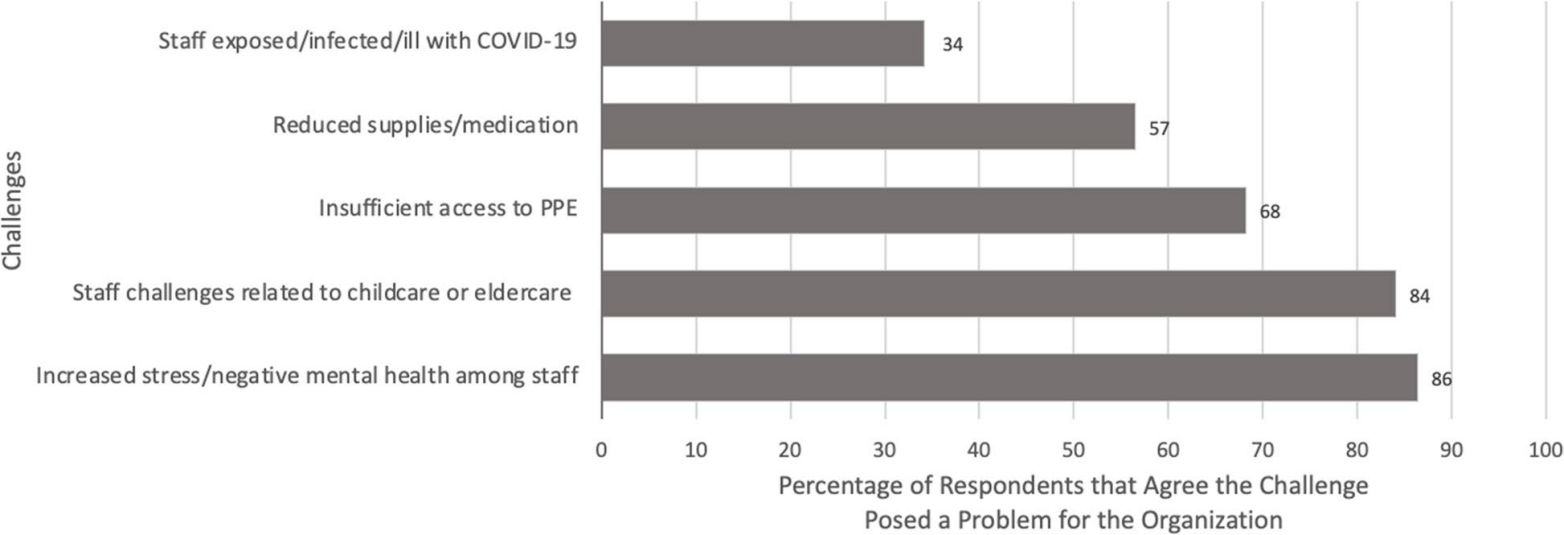
Attitudes on challenges to Pennsylvania OTP operations during the COVID-19 pandemic (*n* = 44)

More than 80% of respondents agreed that increased negative mental health among staff (86%, *n* = 38) and staff childcare and eldercare responsibilities (84%, *n* = 37) were challenges to OTP operations during the pandemic (Fig. 2). Sixty-eight percent of respondents (*n* = 30) agreed insufficient personal protective equipment access was a challenge, 57% (*n* = 25) agreed insufficient supplies and medications was a challenge, and 34% (*n* = 15) of respondents reported staff exposure, infection and illness with COVID-19 was a challenge to operations.

## Discussion

Our study seeks to understand modifications to operations and adoptions of safety measures at Pennsylvania OTPS during the COVID-19 pandemic. Consistent with prior research [17–23], we find that the vast majority of OTPs made at least one modification to services or operations during the pandemic, but service modifications varied by type of services. However, we find that over half of respondents reported not changing the volume of services or moving to virtual services for medications for opioid use disorder, same-day initiation, case management services, drug screening, infectious disease management services, and naloxone distribution. Patients may be reluctant or unable to attend clinical care schedules and meet requirements that were set pre-pandemic, which may cause disruptions to care [25]. Service and operation adaptations should take into account the preferences and needs of patients, which are still being characterized in the literature [21, 25–27].

Our study elucidates that OTPs readily adopted safety protocols. However, OTPs were understandably more likely to adopt safety protocols that did not require significant funding, such as limiting the number of people entering the site, enforcing social distancing, posting information and increasing sanitation. Programs were less likely to make physical alterations to space, refer patients to other providers, or stagger visits. In addition, only one-third of respondents were able to provide most of their staff with N95 masks and 68% reported challenges related to insufficient access to personal protective equipment. While emergency funding was made available to increase access to substance use disorder treatment during the pandemic, [28] these findings speak to the need for emergency funding to ensure that OTP providers in Pennsylvania can adopt protocols to deliver these services safely. OTPs limited ability to quickly adopt safety measures that require additional funding during this initial phase of the pandemic may also inform future emergency planning in subsequent phases of the COVID-19 pandemic and future public health disasters that lead to major disruption in health services, which are predicted to increase in the coming decades due to climate change [29, 30].

Research on OTP operations during prior emergency situations may inform the interpretation of these findings. Studies on OTP operations during Hurricane Sandy and the immediate aftermath of the September 11^th^ attack in New York City found disruptions to care and increased relapse among patients [31, 32]. The rapid rise in overdose deaths in 2020 and the reduced number of clients served by OTPs in our survey allude to similar disruptions in care during the COVID-19 pandemic. Qualitative studies on OTP operations during hurricanes find that providers report that having robust emergency planning prior to a public health disaster, with a focus on communication, transportation, and policies to enhance guest dosing and take-home provisions would reduce burden on OTPs [33–35]. These findings in the context of the inconsistent application of safety protocols found in this study speak to the need for OTPs to have a distinct resource and regulatory body from whom they can receive clearly-communicated safety protocols and procedure updates. It also highlights the difficulty in communicating rapidly changing safety information and providing safety equipment at a mass scale, particularly for OTPs, which are siloed from the broader U.S. health care system [36, 37]. This separation is a barrier to maintaining and enhancing access to opioid treatment during such disasters, in particular because of the critical emergency preparedness and response that occurs at the health system level [38, 39]. Lessons learned from prior emergency situations are particularly applicable to the results presented in this study which are from earlier phases of the pandemic, but may be less applicable as the pandemic has prolonged and continued to affect long-term operations.

Several studies have documented that during the pandemic there was an increase in negative mental health outcomes in the general population, in part associated with concerns about contracting COVID-19, financial stability, and caregiving challenges [40–44]. Similar psychological, social and material stressors observed during other emergency and disaster events have been associated with drug use and relapse among individuals in substance use disorder treatment [45–48]. These stressors may also explain why almost half of OTPs in this survey reported reductions in the number of clients served, indicating disruptions to care. These challenges experienced in the population more broadly are also reflected by our finding that over 80% of respondents reported that OTPs experienced challenges related to staff’s caregiving responsibilities, stress, and negative mental health. Identifying mechanisms to improve mental health and flexible funding mechanisms to support caregiving and other staff and patient needs is another key future intervention point, to ensure that OTPs in the state can adapt during crises and minimize disruptions to care. Bolstering OTPs’ ability to provide services during the pandemic is particularly important given the link between rapidly rising overdose rates associated with synthetic opioids, against which opioid agonist treatments are powerful protective tools [10].

### Limitations

These results should be taken in the context of several limitations. First, this survey sample is not necessarily representative of all OTPs in the state due to differences between responding and non-responding OTPs within Pennsylvania. For example, approximately a quarter of non-responding OTPs were not-for-profit, compared to 36% of respondents. This survey was also limited to OTPs in a single state and therefore may not be generalizable to OTPs in other states. Pennsylvania has the fifth highest drug overdose death rate in the U.S. and has historically held particularly strict regulations over OTPs. Second, findings are representative of practices in late 2020. Understanding of COVID-19 transmission, safety protocols and practices of OTPs shifted since then and wider vaccine availability has facilitated changes in health care operations. Third, while OTPs reported an overall reduction in the number of clients served, we are unable to ascertain if increases and decreases to the provision of specific services resulted in a different number of clients receiving those services or a different amount of service volume per client.

## Conclusions

Overall, this survey finds that the vast majority of OTPs in Pennsylvania responded to the COVID-19 pandemic by making services and safety modifications. OTPs reported reductions in the number of clients served, difficulties providing staff with personal protective equipment, and challenges related to staff mental health. As the pandemic is still ongoing, addressing these challenges will be key to ensuring that OTP provision of medication treatment is not further disrupted during this unprecedented overdose crisis.

## Supplementary Information

**Additional file 1.** Supplemental Digital Content 1-Survey Instrument.

## Data Availability

The datasets used and/or analysed during the current study are available from the corresponding author on reasonable request.

## Abbreviations

COVID-19: Novel coronavirus 2019
MOUD: Medications for opioid use disorder
OTP: Opioid treatment program

## References

[1] Centers for Disease Control and Prevention. 2021. COVID-19-Your Health [Internet]. [Cited 2021 Apr 18]. Available from: https://www.cdc.gov/coronavirus/2019-nCoV/index.html

[2] Koonin LM, Hoots B, Tsang CA, Leroy Z, Farris K, Jolly B (2020). Trends in the Use of Telehealth During the Emergence of the COVID-19 Pandemic — United States, January–March 2020. MMWR Morb Mortal Wkly Rep.

[3] Raifman J, Nocka K, Jones D, Bor J, Lipson S, Jay J, et al. 2020. COVID-19 US state policy database [Internet]. COVID-19 US state policy database. [Cited 2021 Jul 1]. Available from: www.statepolicies.com

[4] Volkow ND (2020). Collision of the COVID-19 and Addiction Epidemics. Ann Intern Med.

[5] Alexander GC, Stoller KB, Haffajee RL, Saloner B. 2020. An Epidemic in the Midst of a Pandemic: Opioid Use Disorder and COVID-19. Ann Intern Med [Internet]. [Cited 2020 May 14]. Available from: https://annals.org/aim/fullarticle/2764311/epidemic-midst-pandemic-opioid-use-disorder-covid-19 10.7326/M20-1141 PMC7138407 32240283

[6] Ahmad F, Rossen L, Sutton P (2020). Provisional drug overdose death counts. National Center for Health Statistics.

[7] Ochalek TA, Cumpston KL, Wills BK, Gal TS, Moeller FG (2020). Nonfatal Opioid Overdoses at an Urban Emergency Department During the COVID-19 Pandemic. JAMA.

[8] Friedman J, Beletsky L, Schriger DL (2021). Overdose-Related Cardiac Arrests Observed by Emergency Medical Services During the US COVID-19 Epidemic. JAMA Psychiatry..

[9] Krawczyk N, Mojtabai R, Stuart EA, Fingerhood M, Agus D, Lyons BC (2020). Opioid agonist treatment and fatal overdose risk in a state-wide US population receiving opioid use disorder services. Addiction.

[10] National Academies of Sciences, Engineering, and Medicine; Health and Medicine Division; Board on Health Sciences Policy; Committee on Medication-Assisted Treatment for Opioid Use Disorder. Medications for Opioid Use Disorder Save Lives [Internet]. Mancher M, Leshner AI, editors. Washington (DC): National Academies Press (US); 2019 [cited 2020 May 14]. (The National Academies Collection: Reports funded by National Institutes of Health). Available from: http://www.ncbi.nlm.nih.gov/books/NBK538936/30896911

[11] World Health Organization, International Narcotics Control Board, United Nations Office on Drugs and Crime, editors (2009). Guidelines for the psychosocially assisted pharmacological treatment of opioid dependence.

[12] Bahji A, Cheng B, Gray S, Stuart H (2019). Reduction in mortality risk with opioid agonist therapy: a systematic review and meta-analysis. Acta Psychiatr Scand.

[13] National Institute on Drug Abuse. 2018. Medications to Treat Opioid Use Disorder Research Report [Internet]. [Cited 2021 Mar 17]. Available from: https://www.drugabuse.gov/download/21349/medications-to-treat-opioid-use-disorder-research-report.pdf?v=99088f7584dac93ddcfa98648065bfbe

[14] Institute of Medicine (US) Committee on Federal Regulation of Methadone Treatment; Rettig RA, Yarmolinsky A, editors. Washington (DC): National Academies Press (US); 1995. 25121195

[15] Joudrey PJ, Edelman EJ, Wang EA (2020). Methadone for Opioid Use Disorder—Decades of Effectiveness but Still Miles Away in the US. JAMA Psychiat.

[16] Davis CS, Samuels EA (2020). Opioid Policy Changes During the COVID-19 Pandemic - and Beyond. J Addict Med.

[17] Brothers S, Viera A, Heimer R (2021). Changes in methadone program practices and fatal methadone overdose rates in Connecticut during COVID-19. J Subst Abuse Treat..

[18] Krawczyk N, Maniates H, Hulsey E, Smith JS, DiDomenico E, Stuart EA, et al. 2022. Shifting Medication Treatment Practices in the COVID-19 Pandemic: A Statewide Survey of Pennsylvania Opioid Treatment Programs. J Addict Med [Internet]. [Cited 2022 Mar 16];Publish Ahead of Print. Available from: https://journals.lww.com/10.1097/ADM.0000000000000981 10.1097/ADM.0000000000000981 PMC9653109 35165225

[19] Quiñones DS, Melin K, Roman L, Rodriguez F, Alvarado J, Rodríguez-Díaz CE. 2020. Treating Opioid Use Disorder in Puerto Rico During the COVID-19 Pandemic: Providers’ Leadership Efforts in Unprecedented Times. J Addict Med [Internet]. [Cited 2021 Jul 18];Publish Ahead of Print. Available from: https://journals.lww.com/10.1097/ADM.0000000000000764 10.1097/ADM.0000000000000764 PMC8327765 33229933

[20] Joseph G, Torres-Lockhart K, Stein MR, Mund PA, Nahvi S (2021). Reimagining patient-centered care in opioid treatment programs: Lessons from the Bronx during COVID-19. J Subst Abuse Treat..

[21] Figgatt MC, Salazar Z, Day E, Vincent L, Dasgupta N (2021). Take-home dosing experiences among persons receiving methadone maintenance treatment during COVID-19. J Subst Abuse Treat..

[22] Hunter SB, Dopp AR, Ober AJ, Uscher-Pines L (2021). Clinician perspectives on methadone service delivery and the use of telemedicine during the COVID-19 pandemic: A qualitative study. J Subst Abuse Treat..

[23] Office of the Inspector General, U.S. Department of Health and Human Services. 2020. Opioid Treatment Programs Reported Challenges Encountered During the COVID-19 Pandemic and Actions Taken to Address Them [Internet]. [Cited 2021 Jul 18]. Report No.: No. A-09–20–01001. Available from: https://oig.hhs.gov/oas/reports/region9/92001001.pdf

[24] CDC, National Center for Health Statistics. 2021. Drug Overdose Mortality by State [Internet]. [Cited 2021 Jul 18]. Available from: https://www.cdc.gov/nchs/pressroom/sosmap/drug_poisoning_mortality/drug_poisoning.htm

[25] Krawczyk N, Bunting AM, Frank D, Arshonsky J, Gu Y, Friedman SR (2021). “How will I get my next week’s script?” Reactions of Reddit opioid forum users to changes in treatment access in the early months of the coronavirus pandemic. Int J Drug Policy..

[26] Russell C, Ali F, Nafeh F, Rehm J, LeBlanc S, Elton-Marshall T (2021). Identifying the impacts of the COVID-19 pandemic on service access for people who use drugs (PWUD): A national qualitative study. J Subst Abuse Treat..

[27] Saloner B, Krawczyk N, Solomon K, Allen ST, Morris M, Haney K, Sherman SG. Experiences with substance use disorder treatment during the COVID-19 pandemic: Findings from a multistate survey. Int J Drug Policy. 2002;101:103537. 10.1016/j.drugpo.2021.103537. 10.1016/j.drugpo.2021.103537 PMC8602971 34871945

[28] Substance Abuse and Mental Health Services Administration. 2021. Emergency Grants to Address Mental and Substance Use Disorder During COVID-19 [Internet]. [cited 2021 Jul 18]. Available from: https://www.samhsa.gov/grants/grant-announcements/fg-20-006

[29] Frumkin H, Hess J, Luber G, Malilay J, McGeehin M (2008). Climate Change: the Public Health Response. Am J Public Health.

[30] Haines A, Kovats RS, Campbell-Lendrum D, Corvalan C (2006). Climate change and human health: impacts, vulnerability and public health. Public Health.

[31] Frank B, Dewart T, Schmeidler J, Demirjian A (2006). The Impact of 9/11 on New York City’s Substance Abuse Treatment Programs: A Study of Program Administrators. J Addict Dis.

[32] Pouget ER, Sandoval M, Nikolopoulos GK, Friedman SR (2015). Immediate Impact of Hurricane Sandy on People Who Inject Drugs in New York City. Subst Use Misuse.

[33] McClure B, Mendoza S, Duncan L, Rotrosen J, Hansen H (2014). Effects of Regulation on Methadone and Buprenorphine Provision in the Wake of Hurricane Sandy. J Urban Health.

[34] Matusow H, Benoit E, Elliott L, Dunlap E, Rosenblum A (2018). Challenges to Opioid Treatment Programs After Hurricane Sandy: Patient and Provider Perspectives on Preparation, Impact, and Recovery. Subst Use Misuse.

[35] Elliott L, Benoit E, Matusow H, Rosenblum A (2017). Disaster preparedness among opioid treatment programs: Policy recommendations from state opioid treatment authorities. Int J Disaster Risk Reduct.

[36] Saloner B, Stoller KB, Alexander GC (2018). Moving Addiction Care to the Mainstream — Improving the Quality of Buprenorphine Treatment. N Engl J Med.

[37] O’Connor PG, Nyquist JG, McLellan AT (2011). Integrating Addiction Medicine Into Graduate Medical Education in Primary Care: The Time Has Come. Ann Intern Med.

[38] Rutkow L, Vernick JS, Mojtabai R, Rodman SO, Kaufmann CN (2012). Legal challenges for substance abuse treatment during disasters. Psychiatr Serv Wash DC.

[39] Kaji AH, Koenig KL, Lewis RJ (2007). Current Hospital Disaster Preparedness. JAMA.

[40] McGinty EE, Presskreischer R, Anderson KE, Han H, Barry CL (2020). Psychological Distress and COVID-19–Related Stressors Reported in a Longitudinal Cohort of US Adults in April and July 2020. JAMA.

[41] Ettman CK, Abdalla SM, Cohen GH, Sampson L, Vivier PM, Galea S (2021). Low assets and financial stressors associated with higher depression during COVID-19 in a nationally representative sample of US adults. J Epidemiol Community Health.

[42] Holingue C, Kalb LG, Riehm KE, Bennett D, Kapteyn A, Veldhuis CB (2020). Mental Distress in the United States at the Beginning of the COVID-19 Pandemic. Am J Public Health.

[43] Holingue C, Badillo-Goicoechea E, Riehm KE, Veldhuis CB, Thrul J, Johnson RM (2020). Mental distress during the COVID-19 pandemic among US adults without a pre-existing mental health condition: Findings from American trend panel survey. Prev Med..

[44] Patrick SW, Henkhaus LE, Zickafoose JS, Lovell K, Halvorson A, Loch S (2020). Well-being of Parents and Children During the COVID-19 Pandemic: A National Survey. Pediatrics..

[45] Cales RH, Cales SC, Shreffler J, Huecker MR. The COVID-19 pandemic and opioid use disorder: expanding treatment with buprenorphine, and combining safety precautions with telehealth. J Subst Abuse Treat. 2022;133:108543. 10.1016/j.jsat.2021.108543. 10.1016/j.jsat.2021.108543 PMC8233546 34210567

[46] Dasgupta N, Beletsky L, Ciccarone D (2018). Opioid Crisis: No Easy Fix to Its Social and Economic Determinants. Am J Public Health.

[47] North CS, Ringwalt CL, Downs D, Derzon J, Galvin D (2011). Postdisaster Course of Alcohol Use Disorders in Systematically Studied Survivors of 10 Disasters. Arch Gen Psychiatry.

[48] Lowe SR, Sampson L, Young MN, Galea S (2017). Alcohol and Nonmedical Prescription Drug Use to Cope With Posttraumatic Stress Disorder Symptoms: An Analysis of Hurricane Sandy Survivors. Subst Use Misuse.

