# Supplementary material for: Opioid treatment program safety measures during the COVID-19 pandemic: a statewide survey
Source: BMC Health Serv Res. 2022 Mar 30;22:418. doi: 10.1186/s12913-022-07832-7 (PMC8965537; doi:10.1186/s12913-022-07832-7)
Supplement: Supplementary file 1 — Additional file 1. Supplemental Digital Content 1-Survey Instrument. [file 12913_2022_7832_MOESM1_ESM.pdf]

Supplemental Digital Content 1-Survey Instrument

Start of Block: I. Organization and services prior to COVID-19

Organization and services prior to COVID-19
This section will ask you questions about your program and services offered before the COVID-19 pandemic. All responses will be kept confidential and presented in aggregate. No individual responses will be reported to the PA Department of Drug and Alcohol Programs (DDAP).

Which of the following best describes your organization type? (check one)

○ a. Non-profit organization (1)

○ b. For-profit organization (2)

○ c. City or state-run clinic or organization (3)

○ d. University or medical organization-affiliated program (4)

○ e. Other: (5) ____

Approximately how many full-time equivalent staff were employed by your organization prior to COVID-19? (number)

____

Which of the following services did your organization provide ***<u>prior to COVID-19</u>****? (check all that apply)*

- ☐ a. Overdose prevention/education (4)
- ☐ b. Naloxone (Narcan®) distribution (5)
- ☐ c. Syringe services program (6)
- ☐ d. Distribution of fentanyl testing/test strips (7)
- ☐ e. Distribution of safer sex items (e.g., male and insertive condoms, lubricant) (8)
- ☐ f. Distribution of PrEP (9)
- ☐ g. Drug checking machine (e.g., Brueker Alpha) (10)
- ☐ h. Safe injection 101 (11)
- ☐ i. Peer services/street outreach for harm reduction (12)
- ☐ j. Medications for opioid use disorder (methadone, buprenorphine, or Vivitrol®) (13)
- ☐ k. Individual counseling/behavioral therapy (14)
- ☐ l. Group counseling/behavioral therapy (15)
- ☐ m. Peer recovery coaches (16)
- ☐ n. Self help or 12-step programs (e.g. Narcotics Anonymous, Alcoholics Anonymous, SMART Recovery) (17)
- ☐ o. Urine/saliva drug screening (18)

- [ ] p. Walk-in/same-day treatment initiation (19)
- [ ] q. On-site case management/social services (assistance with food, housing, employment, insurance) (20)
- [ ] r. Referral out to case management/social services (21)
- [ ] s. Housing units provided by the program (22)
- [ ] t. Religious or spiritual counseling/services (23)
- [ ] u. Childcare (24)
- [ ] v. Domestic violence-related services (25)
- [ ] w. Educational services/job training/GED (26)
- [ ] x. Drop-in area/safe place to hang out (30)
- [ ] y. Wound care (27)
- [ ] z. Infectious disease (including HIV and STI) testing and/or treatment (28)
- [ ] aa. Special programming for youth (less than age 25) (29)
- [ ] bb. Special programming for justice-involved individuals (e.g. drug court, parole/probation) (31)
- [ ] cc. Special programming for pregnant and/or early parenting (32)

Page Break

Display This Question:

If Which of the following services did your organization provide prior to COVID-19? (check all that... = z. Infectious disease (including HIV and STI) testing and/or treatment

INFECTIOUS_DISEASE *Which of the following infectious disease testing and/or treatment services did your organization provide* ***prior to COVID-19****?* *(check all that apply)*

	Testing (1)	Treatment (2)
a. HIV/AIDS (296)	▢	▢
b. Hepatitis C (HCV) (297)	▢	▢
c. Hepatitis A (HAV) (298)	▢	▢
d. Hepatitis B (HBV) (299)	▢	▢
e. Gonorrhea (300)	▢	▢
f. Chlamydia (301)	▢	▢
g. Syphilis (302)	▢	▢
h. Tuberculosis (303)	▢	▢

Page Break

Display This Question:

If Which of the following services did your organization provide prior to COVID-19? (check all that... = j. Medications for opioid use disorder (methadone, buprenorphine, or Vivitrol®)

MEDICATIONS
Which of the following medications for opioid use disorder services did your organization offer **prior to COVID-19**? (check all that apply)

- ☐ a. Methadone maintenance (1)
- ☐ b. Methadone as detox/taper (2)
- ☐ c. Buprenorphine maintenance (3)
- ☐ d. Buprenorphine as detox/taper (4)
- ☐ e. Extended-release naltrexone (Vivitrol®) maintenance (5)
- ☐ Short term Vivitrol use (6)

Display This Question:

If Which of the following medications for opioid use disorder services did your organization offer p... = c. Buprenorphine maintenance

Or Which of the following medications for opioid use disorder services did your organization offer p... = d. Buprenorphine as detox/taper

BUPE_MODE *Please indicate the modes by which buprenorphine was offered at your organization **prior to COVID-19** (check all that apply)*

- ☐ a. Prescribed buprenorphine by a waivered clinician that patients fill at a community pharmacy (1)
- ☐ b. Buprenorphine that is dispensed onsite (e.g., through a medication window) (2)

Page Break

TAKEHOME_PRIOR *What proportion of patients at your clinic do you estimate were receiving methadone take-home doses of* ***3 days or more*** ***prior to COVID-19****?*

- ☐ 0% (1)
- ☐ 1-25% (2)
- ☐ 26-50% (3)
- ☐ 51-75% (4)
- ☐ 76-100% (5)
- ☐ If you know the exact percentage of patients, please fill in here (6) ____

METHADONE_SAFETY_PRE *Which of the following strategies to reduce diversion and/or adverse events for patients receiving* ***methadone take-homes*** *did you have in place* ***prior to COVID-19****? (check all that apply)*

- ☐ a. Requiring medication lock boxes (1)
- ☐ b. Requiring return of empty bottles (2)
- ☐ c. Random call-backs of medication (3)
- ☐ d. Regular drug screening (6)
- ☐ e. Random drug screening (4)
- ☐ f. None of the above (5)

BUPE_SAFETY_PRE Which of the following strategies to reduce diversion and/or adverse events for patients receiving **buprenorphine take-homes or longer prescriptions** did you have in place **prior to COVID-19?** (check all that apply)

- ☐ a. Requiring medication lock boxes (1)
- ☐ b. Requiring return of empty bottles (2)
- ☐ c. Random call-backs of medication (3)
- ☐ d. Regular drug screening (6)
- ☐ e. Random drug screening (4)
- ☐ f. None of the above (5)

TELEHEALTH

*Which of the following telehealth/telemedicine services did your organization provide **prior to COVID-19**? (check all that apply)*

- ☐ a. Counseling appointments (1)
- ☐ b. Buprenorphine refills/check-ins (2)
- ☐ c. Peer outreach/case management check-ins (3)
- ☐ d Other (5) ____
- ☐ e. None of these (4)

INSURANCE *What insurance types did your organization accept **prior to COVID-19**? (check all that apply)*

- ☐ a. Private insurance (1)
- ☐ b. Medicaid (2)
- ☐ c. Medicare (3)
- ☐ d. Military/VA insurance (4)
- ☐ e. No insurance (e.g., have block grant funding to cover uninsured or sliding scale payment) (5)
- ☐ f. Not applicable - Our services are not billed to insurance (6)

Page Break

CLIENTS SERVED *Please provide your best estimate for the distribution of your clients for the following demographic characteristics **prior to COVID-19:***

CLIENTS SERVED *How many clients do you estimate you served on a typical day **prior to COVID-19**? (number)*

____

CS_RACE ***Race/ethnicity:***

	None or nearly none (1)	Some (2)	Most (3)	All or nearly all (4)	Don't Know (5)
White (6)	○	○	○	○	○
Black (1)	○	○	○	○	○
Hispanic/Latino (2)	○	○	○	○	○
Other (4)	○	○	○	○	○

CS_GENDE ***Gender:***

	None or nearly none (1)	Some (2)	Most (3)	All or nearly all (4)	Don't Know (5)
Male (1)	○	○	○	○	○
Female (2)	○	○	○	○	○
Transgender (4)	○	○	○	○	○
Gender nonconforming/nonbinary (5)	○	○	○	○	○

CS_ORIENTATION ***Sexual orientation:***

	None or nearly none (1)	Some (2)	Most (3)	All or nearly all (4)	Don't Know (5)
Heterosexual or straight (1)	○	○	○	○	○
Homosexual, gay, or lesbian (2)	○	○	○	○	○
Bisexual (4)	○	○	○	○	○
Other (5)	○	○	○	○	○

CS_AGE ***Age:***

	None or nearly none (1)	Some (2)	Most (3)	All or nearly all (4)	Don't Know (5)
1-17 (1)	○	○	○	○	○
18-25 (2)	○	○	○	○	○
26-45 (4)	○	○	○	○	○
46-65 (5)	○	○	○	○	○
66+ (6)	○	○	○	○	○

CS_EDU ***Education level:***

	None or nearly none (1)	Some (2)	Most (3)	All or nearly all (4)	Don't Know (5)
Less than/some high school (1)	○	○	○	○	○
High school degree (2)	○	○	○	○	○
Some college/university (4)	○	○	○	○	○
College/University degree (5)	○	○	○	○	○

End of Block: I. Organization and services prior to COVID-19

Start of Block: II. Service Changes Following COVID-19

QII **Service Changes Following COVID-19**
This section will ask you questions about how your program currently operates and changes made to your program **after the start of the COVID-19 pandemic.** All responses will be kept confidential and presented in aggregate. No individual responses will be reported to the PA Department of Drug and Alcohol Programs (DDAP).

WORKFORCE_CHANGES *How would you describe changes in the number of full time equivalent staff working at your organization* ***after the COVID-19 pandemic?*** *(choose one)*

○ Greatly reduced (1)

○ Reduced (2)

○ No change (3)

○ Increased (4)

○ Greatly increased (5)

---

14. OTP_WORKFORCE_CH *For which positions have you changed staffing hours since COVID-19? (check all that apply)*

	Reduced (1)	No Change (2)	Increased (3)
Physicians (1)	○	○	○
Nurses (2)	○	○	○
Counselors (3)	○	○	○
Peers (4)	○	○	○
Administrators (5)	○	○	○

CLIENT_VOLUME *How would you describe changes in the volume of clients you serve* ***after the COVID-19 pandemic****? (choose one)*

○ Greatly reduced (1)

○ Reduced (2)

○ No change (3)

○ Increased (4)

○ Greatly increased (5)

COVID_CHANGE *Did your organization modify services due to/in response to the COVID-19 pandemic? (includes any changes in hours, staff, protocols, modes of delivery, safety, etc.)*

○ Yes (1)

○ No (2)

Display This Question:

If Did your organization modify services due to/in response to the COVID-19 pandemic? (includes any... = Yes

DATE_COVID_CHANGE *What date did your organization decide to adjust services in order to reduce patient/staff risk of exposure to COVID-19? __________ [MM/DD/YR]*

____

HOURS_MODIFICATIONS *Have your program's hours of operation per week changed following COVID-19 closures and regulation changes? (choose one)*

- ○ No change (1)
- ○ Suspended our program (2)
- ○ Reduced our hours (3)
- ○ Increased our hours (4)

Page Break

Display This Question:

If Did your organization modify services due to/in response to the COVID-19 pandemic? (includes any... = Yes

And Which of the following services did your organization provide prior to COVID-19? (check all that... = j. Medications for opioid use disorder (methadone, buprenorphine, or Vivitrol®)

MOD_MOUD Service modifications - **Medications for opioid use disorder (methadone, buprenorphine, or Vivitrol®)**
Please note if this service you previously offered has changed after the COVID-19 pandemic

- ▢ No change (11)
- ▢ Changed service schedule (12)
- ▢ Suspended (13)
- ▢ Reduced (14)
- ▢ Increased (15)
- ▢ Switched to virtual/remote (16)

Page Break

Display This Question:

If Did your organization modify services due to/in response to the COVID-19 pandemic? (includes any... = Yes

And Which of the following services did your organization provide prior to COVID-19? (check all that... = k. Individual counseling/behavioral therapy

MOD_COUNSEL Service modifications - **Individual counseling/behavioral therapy**
Please note if this service you previously offered has changed after the COVID-19 pandemic

- ▢ No change (6)
- ▢ Changed service schedule (7)
- ▢ Suspended (8)
- ▢ Reduced (9)
- ▢ Increased (10)
- ▢ Switched to virtual/remote (11)

Page Break

Display This Question:

If Did your organization modify services due to/in response to the COVID-19 pandemic? (includes any... = Yes

And Which of the following services did your organization provide prior to COVID-19? (check all that... = l. Group counseling/behavioral therapy

MOD_GROUP Service modifications - **Group counseling/behavioral therapy**
Please note if this service you previously offered has changed after the COVID-19 pandemic

- ☐ No change (6)
- ☐ Changed service schedule (7)
- ☐ Suspended (8)
- ☐ Reduced (9)
- ☐ Increased (10)
- ☐ Switched to virtual/remote (11)

Page Break

HOME_DELIVERY_MET *Is your organization making the following supplies available via home delivery or pick-up (with social distancing)? (check all that apply)*

	Delivery (1)	Pickup (2)
Methadone (6)	▢	▢

Display This Question:

If Is your organization making the following supplies available via home delivery o = Delivery

And Is your organization making the following supplies available via home delivery o = Pickup

CONTACT_DELIVERY *How do clients get in touch with your organization to schedule pick ups/drop offs (check all that apply)*

▢ Email (1)

▢ Phone (text/call) (2)

▢ Website/Social Media (3)

▢ In person (drop-in) (4)

Page Break

Display This Question:

If Which of the following services did your organization provide prior to COVID-19? (check all that... = j. Medications for opioid use disorder (methadone, buprenorphine, or Vivitrol®)

And Which of the following medications for opioid use disorder services did your organization offer p... = c. Buprenorphine maintenance

BUPRENORPHINE_MODIFI *Which of the following modifications in buprenorphine maintenance treatment has your organization adopted? (check all that apply)*

- ▢ a. Not currently doing buprenorphine intakes (9)
- ▢ b. Longer days supply for buprenorphine (10)
- ▢ c. Suspended or less frequent drug screening (11)
- ▢ d. Relaxed counseling/self-help requirements (12)
- ▢ e. Offering counseling/self-help groups virtually (13)
- ▢ f. Offering video telemedicine appointments for buprenorphine follow-up appointments (14)
- ▢ g. Offering video telemedicine appointments for buprenorphine induction (15)
- ▢ h. Offering telephone appointments for buprenorphine follow-up appointments (video not required) (16)
- ▢ I. Offering telephone appointments for buprenorphine induction (video not required) (17)
- ▢ Other (please describe): (18) ____

Display This Question:

If Which of the following services did your organization provide prior to COVID-19? (check all that... = j. Medications for opioid use disorder (methadone, buprenorphine, or Vivitrol®)

And Which of the following medications for opioid use disorder services did your organization offer p... = a. Methadone maintenance

METHADONE_MODIFICATI *Which of the following modifications in methadone maintenance treatment has your organization adopted? (check all that apply)*

- ▢ a. Not currently doing methadone intakes (9)
- ▢ b. Longer take-home supplies of methadone (10)
- ▢ c. Suspended or less frequent drug screening (11)
- ▢ d. Relaxed counseling/self-help requirements (12)
- ▢ e. Offering counseling/self-help groups virtually (13)
- ▢ f. Offering video telemedicine appointments for methadone follow-up appointments (18)
- ▢ g. Offering telephone appointments for methadone follow-up appointments (video not required) (19)
- ▢ Other (please describe): (20)

Display This Question:

If Which of the following medications for opioid use disorder services did your organization offer p... = a. Methadone maintenance

And Which of the following services did your organization provide prior to COVID-19? (check all that... = j. Medications for opioid use disorder (methadone, buprenorphine, or Vivitrol®)

METHADONE_TAKE_HOMES Which of the following criteria are taken into account for decisions regarding length of allowable methadone take-home supply? (check all that apply)

- ☐ a. Time in program (4)
- ☐ b. Clinical severity of substance use disorder (e.g. length of substance use history, poly-substance use, overdose history) (5)
- ☐ c. Demographic and/or socioeconomic characteristics (e.g. age, housing situation) (6)
- ☐ d. Other (please describe): (7) ____

Display This Question:

If Which of the following medications for opioid use disorder services did your organization offer p... = a. Methadone maintenance

And Which of the following services did your organization provide prior to COVID-19? (check all that... = j. Medications for opioid use disorder (methadone, buprenorphine, or Vivitrol®)

METHADONE_HOME_TXT Please describe in your own words what elements were taken into consideration to determine length of allowable take-home methadone for patients following COVID-19 regulatory changes

____

____

____

____

____

Display This Question:

If Which of the following medications for opioid use disorder services did your organization offer p... = a. Methadone maintenance

And Which of the following services did your organization provide prior to COVID-19? (check all that... = j. Medications for opioid use disorder (methadone, buprenorphine, or Vivitrol®)

TAKEHOME_COVID_14 Following the new regulations for methadone take-homes due to COVID-19, what proportion of patients at your clinic do you estimate began receiving 14-day methadone take-home doses?

- ○ 0% (7)
- ○ 1-25% (8)
- ○ 26-50% (9)
- ○ 51-75% (10)
- ○ 76-100% (11)

Display This Question:

If Which of the following medications for opioid use disorder services did your organization offer p... = a. Methadone maintenance

And Which of the following services did your organization provide prior to COVID-19? (check all that... = j. Medications for opioid use disorder (methadone, buprenorphine, or Vivitrol®)

TAKEHOME_14_TXT If you know the exact number and/or proportion of patients receiving 14-day methadone take-home doses, please fill in here:

- ☐ Number (8) ____
- ☐ Proportion (9) ____

Display This Question:

If Which of the following medications for opioid use disorder services did your organization offer p... = a. Methadone maintenance

And Which of the following services did your organization provide prior to COVID-19? (check all that... = j. Medications for opioid use disorder (methadone, buprenorphine, or Vivitrol®)

TAKEHOME_COVID_28 *Following the new regulations for methadone take-homes due to COVID-19, what proportion of patients at your clinic do you estimate began receiving **28-day methadone take-home doses**?*

- ○ 0% (7)
- ○ 1-25% (8)
- ○ 26-50% (9)
- ○ 51-75% (10)
- ○ 76-100% (11)

Display This Question:

If Which of the following medications for opioid use disorder services did your organization offer p... = a. Methadone maintenance

And Which of the following services did your organization provide prior to COVID-19? (check all that... = j. Medications for opioid use disorder (methadone, buprenorphine, or Vivitrol®)

TAKEHOME_28_TXT If you know the exact number and/or proportion of patients receiving 28-day methadone take-home doses, please fill in here:

- ☐ Number (8) ____
- ☐ Proportion (9) ____

Page Break

DRUG_SCREENING *Following COVID-19, what changes have you implemented to drug screening requirements/practices for patients enrolled in methadone or buprenorphine treatment (check all that apply)*

☐ Temporarily suspended drug screens (1)

☐ Less frequent drug screening in-clinic (2)

☐ Switched from urine to saliva-based drug screening (3)

☐ Require patients to conduct drug screens at home (4)

☐ Outsourced drug screening to another company or provider (5)

☐ No changes to drug screen (6)

Display This Question:

If Which of the following modifications in methadone maintenance treatment has your organization ado... = b. Longer take-home supplies of methadone

METHADONE_SAFETY_POS *Which of the following strategies to reduce diversion and/or adverse events for patients receiving methadone take-homes have you implemented? (check all that apply)*

- ☐ Requiring medication lock boxes (1)
- ☐ Requiring return of empty bottles (2)
- ☐ Random call-backs of medication (3)
- ☐ Regular drug screening (6)
- ☐ Random drug screening (4)
- ☐ None of these strategies (5)

Display This Question:

If Which of the following modifications in methadone maintenance treatment has your organization ado... = b. Longer take-home supplies of methadone

OVERDOSE_SAFETY Following COVID-19, did you offer any of the following services to reduce overdose risk for patients receiving longer methadone take-homes? (check all that apply)

- ☐ Overdose education or resources (1)
- ☐ Naloxone distribution (6)
- ☐ Check-in calls by staff (7)

Display This Question:

If Which of the following modifications in methadone maintenance treatment has your organization ado... = b. Longer take-home supplies of methadone

METHADONE_CONCERNS *Please note how much you agree or disagree with the following perceptions regarding provision of longer take-home methadone doses. (choose one)*

	Strongly Agree (1)	Somewhat agree (3)	Somewhat disagree (5)	Strongly disagree (7)
Take home methadone increases diversion to other people (1)	○	○	○	○
Take home methadone adds a substantial risk that children or other people could accidentally ingest methadone (6)	○	○	○	○
Take home methadone increases risk of overdose (7)	○	○	○	○
Take home methadone is a less burdensome way for patients to get their medication (9)	○	○	○	○
Take home methadone prevents patients from being exposed to COVID-19 at the clinic (10)	○	○	○	○

Display This Question:

If Which of the following modifications in methadone maintenance treatment has your organization ado... = f. Offering video telemedicine appointments for methadone follow-up appointments

Or Which of the following modifications in methadone maintenance treatment has your organization ado... = g. Offering telephone appointments for methadone follow-up appointments (video not required)

METH_TELECONCERN *Please note how much you agree or disagree with the following perceptions regarding methadone-related visits via telemedicine. (choose one)*

	Strongly Agree (1)	Somewhat agree (3)	Somewhat disagree (5)	Strongly disagree (7)
Virtual methadone-related visits increase risk of diversion to other people (1)	○	○	○	○
Virtual methadone-related visits add a substantial risk that children or other people could accidentally ingest methadone (6)	○	○	○	○
Virtual methadone-related visits increase risk of overdose (7)	○	○	○	○
Virtual methadone-related visits are a less burdensome way for patients to get their medication (9)	○	○	○	○
Virtual methadone-related visits prevent patients from being exposed to COVID-19 at the clinic (10)	○	○	○	○

Page Break

Display This Question:

If Which of the following modifications in buprenorphine maintenance treatment has your organization... = b. Longer days supply for buprenorphine

BUPE_SAFETY_POST *Which of the following strategies to reduce diversion and/or adverse events for patients receiving buprenorphine take-homes did you implement? (check all that apply)*

- ☐ Requiring medication lock boxes (1)
- ☐ Requiring return of empty bottles (2)
- ☐ Random call-backs of medication (3)
- ☐ Regular drug screening (6)
- ☐ Random drug screening (4)
- ☐ None of these strategies (5)

Display This Question:

If Which of the following modifications in buprenorphine maintenance treatment has your organization... = f. Offering video telemedicine appointments for buprenorphine follow-up appointments

Or Which of the following modifications in buprenorphine maintenance treatment has your organization... = g. Offering video telemedicine appointments for buprenorphine induction

BUPE_CONCERNS *Please note how much you agree or disagree with the following perceptions regarding provision of buprenorphine via telemedicine. (choose one)*

	Strongly Agree (1)	Somewhat agree (3)	Somewhat disagree (5)	Strongly disagree (7)
Virtual buprenorphine prescribing increases diversion to other people (1)	○	○	○	○
Virtual buprenorphine prescribing adds a substantial risk that children or other people could accidentally ingest buprenorphine (6)	○	○	○	○
Virtual buprenorphine prescribing increases risk of overdose (7)	○	○	○	○
Virtual buprenorphine prescribing is a less burdensome way for patients to get their medication (9)	○	○	○	○
Virtual buprenorphine prescribing prevents patients from being exposed to COVID-19 at the clinic (10)	○	○	○	○

Page Break

OTP_CROWDING *Has your program taken any of the following precautions to reduce crowding among attending patients (check all that apply):*

- ▢ a. Enforced social distancing for patients while clinic/waiting for medication (1)
- ▢ b. Staggering visits/appointment times (2)
- ▢ c. Reduced appointments and programming available (3)
- ▢ d. Expanded or implemented telehealth services (6)
- ▢ e. Other: (5) ____
- ▢ f. No, we did not implement any precautions to reduce crowding due to COVID-19 (4)

Display This Question:

If Has your program taken any of the following precautions to reduce crowding among attending patien... = f. No, we did not implement any precautions to reduce crowding due to COVID-19

OTP_NO_CROWDING *If you have not implemented any precautions to reduce crowding, please indicate why (check all that apply)*

- ☐ a. COVID-19 was not a problem in our area (1)
- ☐ b. Our typical clinic operations were already in line with social distancing recommendations (2)
- ☐ c. Logistical challenges did not allow us to modify our operations due to COVID-19 (3)
- ☐ d. Financial challenges did not allow us to modify our operations due to COVID-19 (4)
- ☐ e. Other: (5) ____

GUIDANCE *How helpful were the following entities in navigating the process of changing your operations or complying with new regulations since COVID-19? (Choose one)*

	Extremely helpful (1)	Very Helpful (2)	Moderately Helpful (3)	Slightly Helpful (4)	Not Helpful at all (5)
Local health department (1)	○	○	○	○	○
State health department (2)	○	○	○	○	○
State agency or regulators (e.g., DDAP) (3)	○	○	○	○	○
Single County Authority (SCA) (4)	○	○	○	○	○
Federal health authority or entity (5)	○	○	○	○	○
Colleagues at other OTP (6)	○	○	○	○	○
Professional associations for OTPs/Addiction providers (e.g. PATOD) (7)	○	○	○	○	○
Non-governmental organization or foundation (8)	○	○	○	○	○

GUIDANCE_TXT Please describe in your own words what assistance was particularly helpful and/or what else you believe could be done in the future to facilitate such transitions?

____

____

____

____

PPE *How much personal protective equipment (PPE) is available to staff and/or clients at your organization? (check all that apply)*

	Staff				Clients			
	None (1)	Some (2)	For most (3)	For all (4)	None (1)	Some (2)	For most (3)	For all (4)
a. N95 masks (1)	○	○	○	○	○	○	○	○
b. Gloves (2)	○	○	○	○	○	○	○	○
c. Surgical and/or cloth masks (3)	○	○	○	○	○	○	○	○

SAFETY_MODIFICATIONS *Which other steps did your program take to manage client and staff risk for COVID-19? (check all that apply)*

- ▢ a. Safety information about COVID-19 posted on flyers/website (8)
- ▢ b. All visitors were required to wear face coverings onsite (9)
- ▢ c. Limited number of people entering site at a time (10)
- ▢ d. Integrated additional sanitation precautions (e.g. wiping surfaces more often) (11)
- ▢ e. Handwashing/sanitizer stations were set up on site (12)
- ▢ f. Screened for COVID-19 symptoms among prior to/upon arrival (13)
- ▢ g. Referred patients elsewhere for services we were not able to provide (14)
- ▢ h. Increased spacing in waiting areas. (15)
- ▢ i. Created triage areas to avoid lines at service access points. (16)
- ▢ j. Added physical barriers, such as plexiglass. (17)
- ▢ k. Other: (18) ____

Page Break

PROGRAM_CHALLENGES Please note to what extent you agree or disagree that each of the following challenges has posed a problem for your organization/staff during COVID-19 *(choose one)*

	Strongly agree (30)	Somewhat agree (31)	Somewhat disagree (32)	Strongly disagree (33)
a. Reduced funding/income due to fewer client visits (4)	○	○	○	○
b. Reduced funding from state or federal programs that cover overhead (5)	○	○	○	○
c. Reduced supplies/medications available (6)	○	○	○	○
d. Staff exposed/infected/ill with COVID-19 (7)	○	○	○	○
e. Not sufficient access to personal protective equipment (PPE) (8)	○	○	○	○
f. Increased stress/negative mental health among staff (9)	○	○	○	○
g. Staff challenges related to childcare or eldercare (10)	○	○	○	○

Display This Question:

If Please note to what extent you agree or disagree that each of the following challenges has posed... = a. Reduced funding/income due to fewer client visits [Strongly agree]

Or Please note to what extent you agree or disagree that each of the following challenges has posed... = a. Reduced funding/income due to fewer client visits [Somewhat agree]

FINANCE_DRIVERS *Which of the following changes due to COVID-19 do you believe are contributing to this worsened financial situation? (check all that apply)*

- ☐ a. Fewer in-person visits for medication receipt (1)
- ☐ b. Fewer counseling/behavioral therapy appointments (2)
- ☐ c. Switch to telemedicine appointments at lower reimbursement rate (3)
- ☐ d. Fewer urine drug screens (4)
- ☐ e. Other: (5) ____

Display This Question:

If Please note to what extent you agree or disagree that each of the following challenges has posed... = a. Reduced funding/income due to fewer client visits [Strongly agree]

Or Please note to what extent you agree or disagree that each of the following challenges has posed... = a. Reduced funding/income due to fewer client visits [Somewhat agree]

Or Please note to what extent you agree or disagree that each of the following challenges has posed... = b. Reduced funding from state or federal programs that cover overhead [Strongly agree]

Or Please note to what extent you agree or disagree that each of the following challenges has posed... = b. Reduced funding from state or federal programs that cover overhead [Somewhat agree]

FINANCE_CLOSING *Please note how much you agree or disagree with the following statement:*I am concerned about the ability of the clinic to financially sustain operations over the long-term

- ○ Strongly Agree (1)
- ○ Somewhat Agree (6)
- ○ Somewhat Disagree (2)
- ○ Strongly Disagree (3)

NEW_REGULATIONS Please note whether you agree with keeping the following changes in regulations and operations permanently after the COVID-19 emergency declaration has ended (check one)

	Strongly Agree (1)	Somewhat agree (3)	Somewhat disagree (5)	Strongly disagree (7)
a. More flexibility on length of take home days for methadone (1)	○	○	○	○
b. Initiation of buprenorphine via telemedicine (requiring video) (2)	○	○	○	○
c. Initiation of buprenorphine via telephone (NOT requiring video) (3)	○	○	○	○
d. Reduced frequency of required drug screening (4)	○	○	○	○
e. Less frequent requirements for counseling/behavioral therapy or self-help groups (5)	○	○	○	○
f. Equal reimbursement for telehealth visits for treatment services as in-person services (6)	○	○	○	○

NEW_REGULATIONS_TXT Please describe in your own words your motivation for keeping or not keeping any of the new regulations for medication for opioid use disorder treatment:

____

____

____

____

Display This Question:

If Did your organization modify services due to/in response to the COVID-19 pandemic? (includes any... = Yes

And Which of the following services did your organization provide prior to COVID-19? (check all that... = a. Overdose prevention/education

MOD_OVERDOSE Service modifications - **Overdose prevention/education**
Please note if this service you previously offered has changed after the COVID-19 pandemic

- ☐ No change (1)
- ☐ Changed service schedule (2)
- ☐ Suspended (3)
- ☐ Reduced (4)
- ☐ Increased (5)
- ☐ Switched to virtual/remote (6)

Page Break

Display This Question:

If Did your organization modify services due to/in response to the COVID-19 pandemic? (includes any... = Yes

And Which of the following services did your organization provide prior to COVID-19? (check all that... = b. Naloxone (Narcan®) distribution

MOD_NALOXONE
Service modifications - **Naloxone (Narcan ®) distribution**
Please note if this service you previously offered has changed after the COVID-19 pandemic

- ☐ No change (6)
- ☐ Changed service schedule (7)
- ☐ Suspended (8)
- ☐ Reduced (9)
- ☐ Increased (10)
- ☐ Switched to virtual/remote (11)

Page Break

Display This Question:

If Did your organization modify services due to/in response to the COVID-19 pandemic? (includes any... = Yes

And Which of the following services did your organization provide prior to COVID-19? (check all that... = c. Syringe services program

MOD_SYRINGE
Service modifications - **Syringe services program**
Please note if this service you previously offered has changed after the COVID-19 pandemic

- ☐ No change (6)
- ☐ Changed service schedule (7)
- ☐ Suspended (8)
- ☐ Reduced (9)
- ☐ Increased (10)
- ☐ Switched to virtual/remote (11)

Page Break

Display This Question:

If Did your organization modify services due to/in response to the COVID-19 pandemic? (includes any... = Yes

And Which of the following services did your organization provide prior to COVID-19? (check all that... = d. Distribution of fentanyl testing/test strips

OD_TESTSTRIP

Service modifications - **Distribution of fentanyl testing/test strips**

Please note if this service you previously offered has changed after the COVID-19 pandemic

- ☐ No change (6)
- ☐ Changed service schedule (7)
- ☐ Suspended (8)
- ☐ Reduced (9)
- ☐ Increased (10)
- ☐ Switched to virtual/remote (11)

Page Break

Display This Question:

If Did your organization modify services due to/in response to the COVID-19 pandemic? (includes any... = Yes

And Which of the following services did your organization provide prior to COVID-19? (check all that... = e. Distribution of safer sex items (e.g., male and insertive condoms, lubricant)

MOD_SAFESEX
Service modifications - **Distribution of safer sex items (e.g., male and insertive condoms, lubricant)**
Please note if this service you previously offered has changed after the COVID-19 pandemic

- ▢ No change (6)
- ▢ Changed service schedule (7)
- ▢ Suspended (8)
- ▢ Reduced (9)
- ▢ Increased (10)
- ▢ Switched to virtual/remote (11)

Page Break

Display This Question:

If Did your organization modify services due to/in response to the COVID-19 pandemic? (includes any... = Yes

And Which of the following services did your organization provide prior to COVID-19? (check all that... = f. Distribution of PrEP

MOD_PREP Service modifications - **Distribution of PrEP**
Please note if this service you previously offered has changed after the COVID-19 pandemic

- ☐ No change (6)
- ☐ Changed service schedule (7)
- ☐ Suspended (8)
- ☐ Reduced (9)
- ☐ Increased (10)
- ☐ Switched to virtual/remote (11)

Page Break

Display This Question:

If Did your organization modify services due to/in response to the COVID-19 pandemic? (includes any... = Yes

And Which of the following services did your organization provide prior to COVID-19? (check all that... = g. Drug checking machine (e.g., Brueker Alpha)

MOD_DRUGCHECK Service modifications - **Drug checking machine (e.g., Brueker Alpha)**
Please note if this service you previously offered has changed after the COVID-19 pandemic

- ☐ No change (6)
- ☐ Changed service schedule (7)
- ☐ Suspended (8)
- ☐ Reduced (9)
- ☐ Increased (10)
- ☐ Switched to virtual/remote (11)

Page Break

Display This Question:

If Did your organization modify services due to/in response to the COVID-19 pandemic? (includes any... = Yes

And Which of the following services did your organization provide prior to COVID-19? (check all that... = h. Safe injection 101

MOD_SAFEINJ Service modifications - **Safe injection 101**
Please note if this service you previously offered has changed after the COVID-19 pandemic

- ▢ No change (6)
- ▢ Changed service schedule (7)
- ▢ Suspended (8)
- ▢ Reduced (9)
- ▢ Increased (10)
- ▢ Switched to virtual/remote (11)

Page Break

Display This Question:

If Did your organization modify services due to/in response to the COVID-19 pandemic? (includes any... = Yes

And Which of the following services did your organization provide prior to COVID-19? (check all that... = i. Peer services/street outreach for harm reduction

MOD_PEER Service modifications - **Peer services/street outreach for harm reduction**
Please note if this service you previously offered has changed after the COVID-19 pandemic

- [ ] No change (6)
- [ ] Changed service schedule (7)
- [ ] Suspended (8)
- [ ] Reduced (9)
- [ ] Increased (10)
- [ ] Switched to virtual/remote (11)

Page Break

Display This Question:

If Did your organization modify services due to/in response to the COVID-19 pandemic? (includes any... = Yes

And Which of the following services did your organization provide prior to COVID-19? (check all that... = m. Peer recovery coaches

MOD_COACH Service modifications - **Peer recovery coaches**
Please note if this service you previously offered has changed after the COVID-19 pandemic

- ☐ No change (6)
- ☐ Changed service schedule (7)
- ☐ Suspended (8)
- ☐ Reduced (9)
- ☐ Increased (10)
- ☐ Switched to virtual/remote (11)

Page Break

Display This Question:

If Did your organization modify services due to/in response to the COVID-19 pandemic? (includes any... = Yes

And Which of the following services did your organization provide prior to COVID-19? (check all that... = n. Self help or 12-step programs (e.g. Narcotics Anonymous, Alcoholics Anonymous, SMART Recovery)

MOD_SELFHELP Service modifications - **Self help or 12-step programs (e.g. Narcotics Anonymous, Alcoholics Anonymous, SMART Recovery)**
Please note if this service you previously offered has changed after the COVID-19 pandemic

- ☐ No change (6)
- ☐ Changed service schedule (7)
- ☐ Suspended (8)
- ☐ Reduced (9)
- ☐ Increased (10)
- ☐ Switched to virtual/remote (11)

Page Break

Display This Question:

If Did your organization modify services due to/in response to the COVID-19 pandemic? (includes any... = Yes

And Which of the following services did your organization provide prior to COVID-19? (check all that... = o. Urine/saliva drug screening

MOD_URINE
Service modifications - **Urine/saliva drug screening**
Please note if this service you previously offered has changed after the COVID-19 pandemic

- ▢ No change (6)
- ▢ Changed service schedule (7)
- ▢ Suspended (8)
- ▢ Reduced (9)
- ▢ Increased (10)
- ▢ Switched to virtual/remote (11)

Page Break

Display This Question:

If Did your organization modify services due to/in response to the COVID-19 pandemic? (includes any... = Yes

And Which of the following services did your organization provide prior to COVID-19? (check all that... = p. Walk-in/same-day treatment initiation

MOD_WALKIN
Service modifications - **Walk-in/same-day treatment initiation**
Please note if this service you previously offered has changed after the COVID-19 pandemic

- ▢ No change (6)
- ▢ Changed service schedule (7)
- ▢ Suspended (8)
- ▢ Reduced (9)
- ▢ Increased (10)
- ▢ Switched to virtual/remote (11)

Page Break

Display This Question:

If Did your organization modify services due to/in response to the COVID-19 pandemic? (includes any... = Yes

And Which of the following services did your organization provide prior to COVID-19? (check all that... = q. On-site case management/social services (assistance with food, housing, employment, insurance)

MOD_CASEMANAGE
Service modifications - **On-site case management/social services (e.g. assistance with food, housing, employment, insurance)**
Please note if this service you previously offered has changed after the COVID-19 pandemic

- ▢ No change (6)
- ▢ Changed service schedule (7)
- ▢ Suspended (8)
- ▢ Reduced (9)
- ▢ Increased (10)
- ▢ Switched to virtual/remote (11)

Page Break

Display This Question:

If Did your organization modify services due to/in response to the COVID-19 pandemic? (includes any... = Yes

And Which of the following services did your organization provide prior to COVID-19? (check all that... = r. Referral out to case management/social services

MOD_REFERRAL
Service modifications - **Referral out to case management/social services**
Please note if this service you previously offered has changed after the COVID-19 pandemic

- ☐ No change (6)
- ☐ Changed service schedule (7)
- ☐ Suspended (8)
- ☐ Reduced (9)
- ☐ Increased (10)
- ☐ Switched to virtual/remote (11)

Page Break

Display This Question:

If Did your organization modify services due to/in response to the COVID-19 pandemic? (includes any... = Yes

And Which of the following services did your organization provide prior to COVID-19? (check all that... = s. Housing units provided by the program

MOD_HOUSING
Service modifications - **Housing units provided by the program**
Please note if this service you previously offered has changed after the COVID-19 pandemic

- ☐ No change (6)
- ☐ Changed service schedule (7)
- ☐ Suspended (8)
- ☐ Reduced (9)
- ☐ Increased (10)
- ☐ Switched to virtual/remote (11)

Page Break

Display This Question:

If Did your organization modify services due to/in response to the COVID-19 pandemic? (includes any... = Yes

And Which of the following services did your organization provide prior to COVID-19? (check all that... = t. Religious or spiritual counseling/services

MOD_RELIGIOUS
Service modifications - **Religious or spiritual counseling/services**
Please note if this service you previously offered has changed after the COVID-19 pandemic

- ▢ No change (6)
- ▢ Changed service schedule (7)
- ▢ Suspended (8)
- ▢ Reduced (9)
- ▢ Increased (10)
- ▢ Switched to virtual/remote (11)

Page Break

Display This Question:

If Did your organization modify services due to/in response to the COVID-19 pandemic? (includes any... = Yes

And Which of the following services did your organization provide prior to COVID-19? (check all that... = u. Childcare

MOD_PARENT
Service modifications - **Childcare-related services**
Please note if this service you previously offered has changed after the COVID-19 pandemic

- ☐ No change (6)
- ☐ Changed service schedule (7)
- ☐ Suspended (8)
- ☐ Reduced (9)
- ☐ Increased (10)
- ☐ Switched to virtual/remote (11)

Page Break

Display This Question:

If Did your organization modify services due to/in response to the COVID-19 pandemic? (includes any... = Yes

And Which of the following services did your organization provide prior to COVID-19? (check all that... = v. Domestic violence-related services

MOD_VIOLENCE
Service modifications - **Domestic violence-related services**
Please note if this service you previously offered has changed after the COVID-19 pandemic

- ☐ No change (6)
- ☐ Changed service schedule (7)
- ☐ Suspended (8)
- ☐ Reduced (9)
- ☐ Increased (10)
- ☐ Switched to virtual/remote (11)

Page Break

Display This Question:

If Did your organization modify services due to/in response to the COVID-19 pandemic? (includes any... = Yes

And Which of the following services did your organization provide prior to COVID-19? (check all that... = w. Educational services/job training/GED

MOD_JOB
Service modifications - **Educational services/job training/GED**
Please note if this service you previously offered has changed after the COVID-19 pandemic

- ☐ No change (6)
- ☐ Changed service schedule (7)
- ☐ Suspended (8)
- ☐ Reduced (9)
- ☐ Increased (10)
- ☐ Switched to virtual/remote (11)

Page Break

Display This Question:

If Did your organization modify services due to/in response to the COVID-19 pandemic? (includes any... = Yes

And Which of the following services did your organization provide prior to COVID-19? (check all that... = x. Drop-in area/safe place to hang out

MOD_DropIn Service modifications - **Drop in area/safe place to hang out**
Please note if this service you previously offered has changed after the COVID-19 pandemic

- ☐ No change (6)
- ☐ Changed service schedule (7)
- ☐ Suspended (8)
- ☐ Reduced (9)
- ☐ Increased (10)
- ☐ Switched to virtual/remote (11)

Page Break

Display This Question:

If Did your organization modify services due to/in response to the COVID-19 pandemic? (includes any... = Yes

And Which of the following services did your organization provide prior to COVID-19? (check all that... = y. Wound care

MOD_WOUND
Service modifications - **Wound care**
Please note if this service you previously offered has changed after the COVID-19 pandemic

- ▢ No change (6)
- ▢ Changed service schedule (7)
- ▢ Suspended (8)
- ▢ Reduced (9)
- ▢ Increased (10)
- ▢ Switched to virtual/remote (11)

Page Break

Display This Question:

If Did your organization modify services due to/in response to the COVID-19 pandemic? (includes any... = Yes

And Which of the following services did your organization provide prior to COVID-19? (check all that... = z. Infectious disease (including HIV and STI) testing and/or treatment

MOD_INFECTIOUS
Service modifications - **Infectious disease (including HIV and STI) testing and/or treatment**
Please note if this service you previously offered has changed after the COVID-19 pandemic

- ▢ No change (6)
- ▢ Changed service schedule (7)
- ▢ Suspended (8)
- ▢ Reduced (9)
- ▢ Increased (10)
- ▢ Switched to virtual/remote (11)

Page Break

Display This Question:

If Did your organization modify services due to/in response to the COVID-19 pandemic? (includes any... = Yes

And Which of the following services did your organization provide prior to COVID-19? (check all that... = aa. Special programming for youth (less than age 25)

MOD_YOUTH
Service modifications - **Special programming for youth (less than age 25)**
Please note if this service you previously offered has changed after the COVID-19 pandemic

- ☐ No change (6)
- ☐ Changed service schedule (7)
- ☐ Suspended (8)
- ☐ Reduced (9)
- ☐ Increased (10)
- ☐ Switched to virtual/remote (11)

Page Break

Display This Question:

If Did your organization modify services due to/in response to the COVID-19 pandemic? (includes any... = Yes

And Which of the following services did your organization provide prior to COVID-19? (check all that... = bb. Special programming for justice-involved individuals (e.g. drug court, parole/probation)

MOD_JUSTICE
Service modifications - **Special programming for justice-involved individuals (e.g. drug court, parole/probation)**
Please note if this service you previously offered has changed after the COVID-19 pandemic

- ▢ No change (6)
- ▢ Changed service schedule (7)
- ▢ Suspended (8)
- ▢ Reduced (9)
- ▢ Increased (10)
- ▢ Switched to virtual/remote (11)

Page Break

Display This Question:

If Did your organization modify services due to/in response to the COVID-19 pandemic? (includes any... = Yes

And Which of the following services did your organization provide prior to COVID-19? (check all that... = cc. Special programming for pregnant and/or early parenting

MOD_Preg Service modifications - **Special programming for pregnant and/or early parenting**
Please note if this service you previously offered has changed after the COVID-19 pandemic

- ☐ No change (6)
- ☐ Changed service schedule (7)
- ☐ Suspended (8)
- ☐ Reduced (9)
- ☐ Increased (10)
- ☐ Switched to virtual/remote (11)

Page Break

Home_Delivery_Other *Is your organization making the following supplies available via home delivery or pick-up (with social distancing)? (check all that apply)*

	Delivery (1)	Pickup (2)
Which of the following services did your organization provide prior to COVID-19? (check all that... = b. Naloxone (Narcan®) distribution Naloxone (1)	☐	☐
Which of the following services did your organization provide prior to COVID-19? (check all that... = c. Syringe services program Sterile syringes (2)	☐	☐
Which of the following services did your organization provide prior to COVID-19? (check all that... = d. Distribution of fentanyl testing/test strips Fentanyl test strips (3)	☐	☐
Which of the following services did your organization provide prior to COVID-19? (check all that... = e. Distribution of safer sex items (e.g., male and insertive condoms, lubricant) Safer sex items (4)	☐	☐
Which of the following services did your organization provide prior to COVID-19? (check all that... = f. Distribution of PrEP Distribution of PrEP (5)	☐	☐

End of Block: II. Service Changes Following COVID-19

Start of Block: III. Lessons Learned: Open Responses

QIII **Lessons Learned: Open Responses**

RE-OPENING_PLANS *If you have modified services due to COVID-19, what plan do you envision for re-opening or returning to usual service provision?*

OTHER_CHALLENGES *Are there any issues and concerns you have about your program and/or clients that we may not have discussed in other questions?*

LESSONS *What are the greatest lessons you've learned throughout this challenge (positive or negative) that you want policymakers/the public to know about?*
